# Topologically protected optical pulling force on synthetic particles through photonic nanojet

**DOI:** 10.1515/nanoph-2023-0740

**Published:** 2024-01-17

**Authors:** Yu-Xuan Ren, Johannes Frueh, Zhisen Zhang, Sven Rutkowski, Yi Zhou, Huade Mao, Cihang Kong, Sergei I. Tverdokhlebov, Wen Liu, Kenneth K. Y. Wong, Bo Li

**Affiliations:** Institute for Translational Brain Research, MOE Frontiers Center for Brain Science, Fudan University, Shanghai, 200032, China; Weinberg Research Center, School of Nuclear Science & Engineering, National Research Tomsk Polytechnic University, 30 Lenin Avenue, 634050 Tomsk, Russian Federation; Institute of Carbon Neutrality, ShanghaiTech University, Shanghai, 201210, China; Department of Optics and Optical Engineering, University of Science and Technology of China, Hefei, 230026, China; Department of Electrical and Electronic Engineering, University of Hong Kong, Pokfulam Road, Hong Kong, SAR 999077, China; Advanced Biomedical Instrumentation Centre, Hong Kong Science Park, Shatin, New Territories, Hong Kong, SAR 999077, China; Department of Neurology, Huashan Hospital, Institute for Translational Brain Research, MOE Frontiers Center for Brain Science, Fudan University, Shanghai, 200032, China

**Keywords:** photonic nanojet, optical pulling force, topological photonics, nanomotor, Janus particle, optical trapping and manipulation

## Abstract

A dielectric microsphere concentrates light into a photonic nanojet (PNJ), and swims towards the near-infrared laser in response to the nanojet-mediated force. In contrast, a Janus particle with an opaque metal layer was thought to be impossible to concentrate light into a stable nanojet. However, the Janus particle may experience optical torque owing to the inhomogeneous composition on both sides even in linearly polarized non-resonant light. Herein, we report on topologically protected PNJ produced by a synthetic Janus particle, and observed the backaction force on the Janus particle. Due to symmetry, the counter-propagating beams can both form PNJ on the respective opposite sides, and pull Janus particles towards respective sources. Furthermore, we unveil that the hysteresis on backaction force with respect to the injection power also exists on synthetic Janus particle compared with their dielectric counterparts. Additionally, the magnitude of the backaction force varies between power increase and decrease stages even with the same laser power. We anticipate that the observation offers great possibilities to pull irregular particles by concentrating light with the particle, and such scheme may be applied for parallel particle manipulation and classification.

## 1 Introduction

Colloids with size comparable to the wavelength of visible light become the building blocks for photonic materials and devices with unique topology and spatial correlation [1], [2]. The inanimate particle can be merely manipulated by the optical radiation pressure force, which can condensate nanoparticles (NPs) that conjugate to biomolecules. Bacteria with flagella move independently according to chemotaxis, and some electricity-producing bacteria can augment electrical current by orders of magnitude once they are optically assembled by laser irradiation [3]. Light-driven micro-swimmer calls for controllable motion in both the direction and the rotation degree of freedom [4], [5]. The photochromic molecules undergo reversible isomerization reactions and the color switching through the photochemical reaction modulates the absorption and scattering forces and induces the reversible motion synchronized with the colorization and decolorization [6]. The optical pulling of particles has arisen hitherto unsuspected importance as the light would attract microparticles rather than push microparticles forward due to the traditional radiation pressure force [7], [8]. Theoretically, the multipole excitation induced forward scattering creates the negative (pulling) force [9]. This can achieve stable transfer of gold-coated hollow glass spheres against the photon flux of a single inhomogeneously polarized beam over tens of centimeters [10]. The momentum topology of a hyperbolic metamaterial with a concave isofrequency contour would excite directional surface plasmon polaritons and increase the forward linear momentum scattered from the elliptical dielectric object, and create broadband optical pulling force [11]. As the polarizability scales with particle volume, nanoparticle exhibit weak trapping force, the optical trapping force can be improved by orders of magnitude through resonance augmented permittivity and polarizability of nanocrystals [12]. Isotropic photocatalytic micromotors experience light-programmable local interactions due to the persistent phoretic flow, and self-organize into nonequilibrium assemblies with light-programmable collective positional and orientational orders [13]. Parallel manipulation of particles can be made possible using structured graphene [14]. The large-scale, long-distance optical pulling of nanoparticle can be demonstrated in the low-refractive index cavity [15], and through supercavitation [16].

Optical heating owing to the photon-to-phonon conversion, as intrinsic loss in metal nanoparticles, can be tuned for opto-thermoelectric nanotweezers [17]. The Marangoni effect suggests programmable swimming actuators that execute multidirectional wavelength-dependent motions [18], [19]. The Janus particle with a carbon coating can absorb light energy and produce a heat gradient to efficiently recruit titanium dioxide microparticles for reconfigurable colloidal assembly [1]. The light-controlled temperature field assembles the colloidal particles and organizes them into complex reconfigurable colloidal matter in the presence of surfactant [20]. The optical force gathers the plasmonic nanoparticles on the wall of glass cuvette to form a decorated cavity, which produces acoustic streaming [21], similar photoacoustic microfluidic pump can even take place by illuminating a gold plate inside the suspension [22]. The rotation speed and direction of a nanoscale plasmonic motor are directly maneuvered by light through tuning the incident wave frequency [23]. Once the plasmonic layer is coated on half of the surface of a dielectric particle, the particle becomes a Janus particle. Janus particle characterizes with heterogeneous composition, and produces anisotropic heating [24], such particle with two distinguishable physical properties can be manipulated by opto-electrophoresis [25], [26]. The surface plasmons excited by a radially polarized beam can enhance the trapping ability of metallic nanoparticles [27]. The plasmonic Janus particle was demonstrated as a microscale elevator in response to incident light of variable power owing to photothermally mediated force [26]. In contrast to anisotropic nanoparticles that move along arbitrary trajectories in response to light, the polarizability of the Janus particle can be tailored through coating thickness [28].

It was generally thought to be incapable of forming a PNJ with Janus particle, since the plasmonic coating localizes light near the surface and apparently degrades the PNJ shape. Our previous simulation suggests that it is possible to concentrate light into an asymmetric, wavelength-tunable PNJ [29], [30]. Although the nanojet is asymmetric at non-resonant IR wavelengths for the Janus cap apex aligned perpendicular to light polarization, it is still possible to form a symmetric and stable nanojet by proper choice of the orientation of the cap apex. Full-phase calculation of the optical torque on Janus particle suggests that these orientations are topologically protected. Such an ability offers the possibility to pull Janus particles through heat-mediated backaction energized by the PNJ. Although a Janus particle experiences rotational Brownian motion in aqueous environment, the Janus particle naturally chooses topologically protected orientations and will form PNJ to generate a self-driven pulling force. Here, we focus on the optical pulling of synthetic particles, including both the homogeneously coated and Janus particles, and demonstrate the pulling of synthetic particles with counter-propagating beams. Our work may excite the research on optical pulling of irregular particles under topological protection, and the application of synthetic particles on nanophotonics, nanomotor and nanobiology.

## 2 Results

### 2.1 Optical torque mediated PNJ through Janus particle

**Figure 1:**
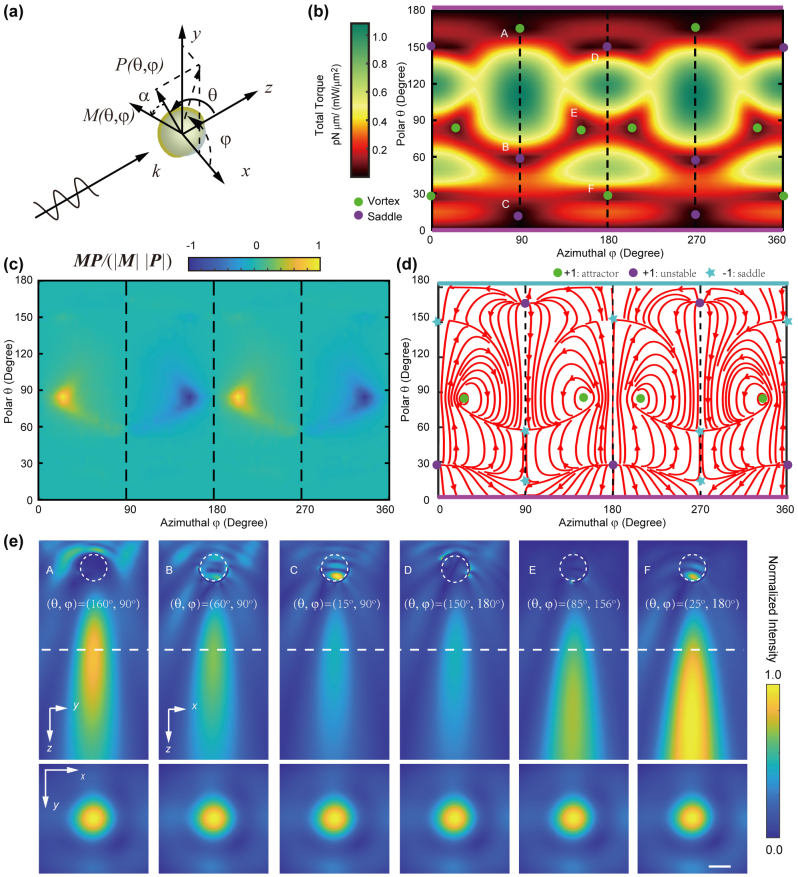
Optical torque on Janus particle. (a) A linearly polarized plane wave $E_{0}\hat{x}$ exerts optical force and torque on a Janus particle. The Janus cap orientation is completely determined by angles *θ*, *φ* with respect to light source. (b) The magnitude of torque and (c) the cosine of the angle between the torque and the cap, for all possible orientations at light wavelength of *λ* = 1550 nm. (d) Stream lines of the ***N*** = ***M*** × ***P*** vector govern the evolution of the particle orientation. Rotational equilibria are the vortex centers at the vector field ***N***. (e) The sideview (upper row) and transverse (lower row) profiles of the PNJ for the orientations labeled in (b). The dashed line is 3 μm away from the particle center. Scale bar for all panels in (e) is 800 nm.

Linearly polarized light can induce a dipole in an irregular particle, and the dipole tends to align the particle’s long axis with light polarization [31]. The Janus particle also experiences torque in the linearly polarized light field due to asymmetry. In the simulation, a linearly polarized plane wave with $E=\hat{x}E_{0}\exp\left(ik_{0}z-i\omega t\right)$ illuminates on a Janus particle of a silica core coated with a thin layer of gold (Figure 1a). The optical force (*F*) and torque (*M*) on the particle can be calculated by integrating the Maxwell stress tensor *T* along a closed boundary *∂S* encircling the particle. Their expressions are given by [32],

(1) $$F=\underset{\partial S}{\int }T\cdot \hat{n}dA,$$

(2) $$M=\underset{\partial S}{\int }r\times \left(T\cdot \hat{n}\right)dA,$$

where the stress tensor is defined as $T_{ij}=\epsilon_{m}\left(E_{i}E_{j}-\frac{1}{2}{\left|E\right|}^{2}\delta_{ij}\right)+\frac{1}{\mu_{0}}\left(B_{i}B_{j}-\frac{1}{2}{\left|B\right|}^{2}\delta_{ij}\right)$ , $\hat{n}$ is the unit vector normal to the enclosing surface, and *ϵ*_*m*_ is the permittivity of the surrounding medium. The light scattering depends strongly on particle orientation, i.e., the plasmonic half coating. The orientation of Janus particle is completely determined by angles of the plasmonic half coating apex (Point P), *θ*, *φ*, which correspond to the polar (w.r.t. $\hat{z}$ ) and the azimuthal angle (w.r.t. $\hat{x}$ ), respectively [33]. Figure 1a exemplifies the coordinates for a 1 μm particle covered with a gold coating (60 nm thick).

The spectral property of synthetic particles depends strongly on the dielectric function of the metal layer. The Janus particle exhibits distinct physical properties on both sides. Here, we consider a dielectric microparticle with a thin layer of gold coating on half of the hemisphere. For the metal layer, the general Drude permittivity describes the response of electron oscillation with respect to the light field,

(3) $$\varepsilon \left(\omega \right)=\varepsilon_{1}+\varepsilon_{2}=\varepsilon_{\infty }-\frac{\omega_{p}^{2}}{\omega^{2}+i\gamma \omega },$$

where *ω* is the angular frequency of light, *ω*_*p*_ is the plasmon frequency, *ɛ*_*∞*_ is a corrective constant for the background electron screening at high frequency, and *γ* represents the scattering frequency of the electron. Light energy would be trapped in the near field [34] characterized by skin depth *δ* = *λ*/2*πk*, and the imaginary part of the refractive index, $k={\left(-\varepsilon_{1}/2+\sqrt{\varepsilon_{1}^{2}+\varepsilon_{2}^{2}}/2\right)}^{1/2}$ . For instance, the dielectric constant at *λ* = 1.55 μm is *ɛ* = −115.13 + 11.26i, and the skin depth is *δ* = 23 nm for gold. The scattered electric and magnetic fields are computed by a finite element solver at the wavelength of *λ* = 1.55 μm in water (*n* = 1.33). Note that, in both the simulation and experiment, the thickness of the gold layer is much greater than the penetration depth across the full visible and NIR spectrum, thus light cannot directly penetrate through the gold coating layer.

To calculate the torque, the stress tensor was integrated on a closed surface surrounding the particle. Permittivity of gold in the finite element simulation is obtained from [35]. Here, the orientation of the Janus cap is defined as the particle director *P*, which can be fully determined by the combination of the polar angle *θ* and the azimuthal angle *φ* [Figure 1(a)]. The stress and torque are calculated for all possible values of *θ*, *φ* in steps of 5°. The magnitude of torque for all possible particle orientations is shown in Figure 1(b). The phase space $\left(\theta ,\varphi \right)$ is split into four symmetric regions of $\left[\gamma ,\gamma +90^{◦}\right)$ for *γ* = 0°, 90°, 180°, 270°. These four regions are closely related due to symmetries of both the Janus particle and the polarization of incident field ( $E=E_{0}\hat{x}$ ) [33]. Due to symmetry, we only mark A∽F in a single region [Figure 1(b)].

The darkest regions in Figure 1(b) show local minimum of total torque, and all the vortices (saddles) are marked with green (purple) dots. Among these orientations, there are four (green dots, for *θ* = 85°, and *φ* = 25°, 155°, 205°, and 335°) that correspond to stable rotational equilibria. These stable points are along the high-symmetry direction with particle orientation vector ***P***. The rotational equilibria can be corroborated by dynamics simulation using translational and rotational Langevin equations [36]. The Janus particle may jump among attractor basins in presence of translational and rotational Brownian motion.

In general, the optical torque ***M*** on the Janus particle does not align with the particle orientation ***P***, we further define the angle between the torque ***M*** and the particle orientation ***P*** as *α*. Figure 1(c) shows the map of $\cos\left(\alpha \right)=M\cdot P/\left|M\right|\left|P\right|$ over all possible particle orientations. The four directions marked with green dots in Figure 1(b) relate to the case when the external torque is precisely aligned (or anti-aligned) with the particle orientation [33]. These four rotationally stable points correspond to a spinning particle (green dots in Figure 1b) with fixed angular momentum under the illumination of linearly polarized plane wave light. This further corroborates the four rotational equilibria in Figure 1(b). The evolution of the apex angle of the Janus particle follows, d ***P***/d*t* = 1/*c*_rot_ ***M*** × ***P***. The evolution of Janus orientation can be well understood on the streamlines of the vector field ***N*** ≡ ***M*** × ***P*** (Figure 1d). All the vortices in the streamline map are labeled in one of these markers, green dots for attractors, purple dots for unstable points, and light blue star for saddle points. The streamlines (Figure 1d) show the same steady orientations consistent with the torque map in Figure 1(b). The induced optical torque at four rotational equilibria is either parallel or anti-parallel to Janus cap direction (Figure 1c). At the Janus orientation for attractors, the Janus particle spins in a steady-state in the light field while the photons possessing zero angular momentum. Physical explanation originates from the difference in coupling the asymmetric Janus particle to two circularly polarized components which linearly polarized beam can be decomposed into.

We apply topology to understand the steady-state attractor. The rotational equilibria are the vortex centers of the vector field ***N*** in the $\left(\theta ,\varphi \right)$ space. These vortices are characterized by their topological charge [37],

(4) $$q=\frac{1}{2\pi }\underset{C}{\oint }dl\cdot \nabla_{l}\gamma \left(l\right),\;q\in Z,$$

Here, *l* is the spherical line element, $\gamma \left(l\right)=\arg\left[N_{\varphi }\left(l\right)+iN_{\theta }\left(l\right)\right]$ is the angle of vector ***N***, and ***C*** is a simple path on the *θ*, *φ* surface that goes counterclockwise around the vortex center. Because the torque vector returns to itself after a closed loop, the overall change must be an integer multiple of 2π; consequently, *q* is an integer. Figure 1(d) shows the position and the charge of all vortices for the wavelength *λ* = 1550 nm.

The topology and the symmetry dictate the properties of the streamlines and the attractors [33]. The topological attractors (green dots) and the unstable extremum points at the pole with *θ* = 0° (horizontal purple line, the gold cap faces away from the light source) have a charge of +1, while saddle points (light blue stars and pole with *θ* = 180°) possess a charge of −1. Close inspection suggests that the sum of all topological charges within the complete phase space $\left(\theta ,\varphi \right)$ equals to 2, i.e., $\sum_{i}q_{i}=2$ , consistent with a Euler characteristic of 2 for the topology of a sphere. The vector ***N*** directs “inward” at every point on a contour enclosing the attractor. Thus, a stable attractor has a charge of +1. Under the action of optical torque, the particle orientation will not escape the enclosing region. Briefly, Janus particles preferentially choose stable orientation that the torque is aligned with the Janus cap director.

The combination of phase-space topology and particle asymmetry offers a powerful degree of freedom in designing a nanoparticle motor for optomechanical applications [33]. The topological attractors correspond to steady-state particle spinning in the plane-wave angular-momentum-free light field. The Janus orientation corresponding to the vortex [marked by green dots in Figure 1(b) and (d)] will eventually change when the Janus cap thickness or the Janus core size is altered. As the orientation changes, the Janus particle may partially concentrate light into asymmetric nanojet, although it can focus the beam into nearly perfect nanojet at some unique orientations. At orientations other than the vortex, the Janus particle keeps spinning and focuses the beam into an orientation dependent jet (either asymmetric or symmetric). In effect, the time averaged light field at the rear side of the Janus particle is homogenized. The orientation of Janus particle will eventually transit to the center of the vortex, such that the torque direction is aligned or anti-aligned with the Janus cap direction. This implies that the spatial distribution of the physical features of the Janus particle won’t change even the particle is spinning. Therefore, the focus field will keep constant as a single time-invariant nanojet.

The stable attractors in the relevant region of the configuration space are topologically protected. Therefore, we inspect the scattering field for the Janus particle at those attractor orientations. Considering the polarization symmetry, we performed FDTD simulation to map the electromagnetic field distribution of the light scattered around a Janus particle with equilibrium orientations marked by letters in Figure 1(b) within one of the four symmetric regions, i.e., $\left[90^{◦},180^{◦}\right)$ . Although the plasmonic coating partially blocks the beam, the Janus particle creates nearly perfect PNJ for each of the orientations as demonstrated in Figure 1(e). The top row shows the sideview profiles, and bottom row demonstrates the transverse profiles at the location marked by the dashed line (top row) [Figure 1(e)]. The dashed circles label the position of the dielectric silica dioxide with a diameter of 1 μm, while the hemispherical shell is marked by the incomplete black curve, and the thickness of the hemispherical gold coating is 60 nm.

In Figure 1(b), we label the vortex and saddle points in the torque map. In general, they fall into two groups: vortex (A, E, F) and saddle point (B, C, D). At the topological vortex, the Janus particle forms a stronger nanojet (A, E, F in Figure 1e). At Points A and F, the Janus particle orientation is not stable. To inspect the beam focus at those orientations (A–F), we further calculate the sideview profiles for the orientations marked with letters in the torque map (Figure 1b). The incident light is polarized along the ***x*** direction and propagates along the ***z*** direction. The sideview for Point A is along the ***yz*** direction (perpendicular to the light polarization), while Points B–F are displayed along ***xz*** direction (aligned with beam polarization). The scale bar for all the transverse and longitudinal profiles is 800 nm. Only the vortex (Point E in Figure 1b) can support stable nanojet. At Point E (attractor), the particle spins around the symmetric axis, equivalent to a time-invariant orientation; therefore, the Janus particle can generate time-invariant stable nanojet despite spinning. At the saddle points, the Janus particles would rotate and find themselves the most stable orientation.

### 2.2 Pulling synthetic Janus particles with PNJ

**Figure 2:**
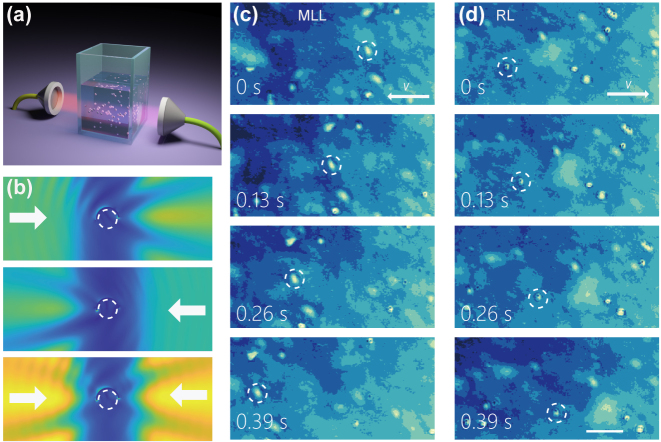
The pulling of synthetic Janus particles. (a) Schematics of the counter-propagating beam setup. (b) The non-resonant IR beams illuminate from opposite sides of the Janus particle, and create respective PNJ. The intensity of each PNJ is displayed in a log scale, while the orientation of the Janus coating is (85°, 156°), corresponding to Point E in Figure 1(b). (c) The video snapshots of Janus particle pulled to the left beam with stronger power of 530 mW in presence of right beam of 210 mW. The average speed for the Janus particle labeled in dashed circle is 0.54 mm/s. (d) The left beam power attenuates to 140 mW, while the Janus particles move towards to the right beam with power of 430 mW at an average speed of 0.14 mm/s. MLL, mode-locked laser; C, cuvette; RL, Raman laser. Scale bar: 50 μm.

The synthetic particles were manipulated by a visible beam through bubble generation [38], [39]. Hence, the pulling of the synthetic particles has not been demonstrated in the near infrared laser beams. To this end, we apply a custom-built counter-propagating beam platform to pull the synthetic particles in the suspension. The all-fiber mode-locked laser (MLL) pumped by a 976-nm laser diode supplies pulses with a repetition rate of 44.5 MHz [40]. The output was amplified with a commercial L-band erbium-doped fiber amplifier (EDFA, bandwidth 1570–1605 nm, IPG photonics) with a final central wavelength of 1576 nm [41]. The counter-propagating beams shine the dielectric suspension from the opposite side (see schematics in Figure 2(a)). The MLL pulls the particles to the left side, while a continuous wave Raman laser (RL, Keopsys Industries) with center wavelength of 1.65 μm shine the suspension from the right hand side [42]. Orthogonal to the counter-propagating beams, a separate visible light from an LED (M530L3, Thorlabs) illuminates the sample, and the sample images were recorded using a bright-field microscope with an objective lens (Olympus, 20×, NA = 0.45), and a CMOS camera (DC1645c-HQ, Thorlabs). The video series were captured at a speed of 20–50 fps and stored for further analysis. The particles in the video series were evaluated using a particle-tracking algorithm to determine the motion trajectories and instantaneous speeds [43]. The backaction force is balanced and evaluated by the fluidic drag force, i.e., *F* = 6*πηrv*, where *η* is the fluid viscosity, *r* and *v* are the radius and speed of the microspheres, respectively.

Janus microparticles offer significant advantages compared to homogeneous particles, especially in the field of drug delivery, due to their symmetry breaking properties [44], [45]. The utilization of these particles allows for controlled optical steering and driving through complex matrices, which makes them very interesting systems for optical control and manipulation [46]. Silicon dioxide (SiO_2_) microparticles with a diameter of (5.00 ± 0.35) µm were purchased from Sigma-Aldrich (Type 44054, 5 % solids). The particles were dispersed in ultrapure water and used directly as dielectric reference particles. For the Janus microparticles, the silicon dioxide microparticles are assembled into a monolayer on top of a glass slide (H878, Carl Roth). After drying, the monolayer of microparticles is coated with 100 nm thick gold through magnetron sputtering [47]. Thereafter, the particles are scratched with a sharp blade from the glass slide and dispersed in ultrapure water by ultrasonication. Such thickness of gold layer (100 nm) is much greater than light penetration depth, thus light would not directly transmit through the plasmonic layer.

We also synthesized decorated microparticles through layer-by-layer approach to coat silicon dioxide microparticles with homogeneous surface decoration with gold nanoparticles [48]. A monolayer of polyethylenimine (PEI, 750 kDa, No. 181978, Merck) assembles on negatively charged silicon dioxide microparticles dispersed within in de-ionized water. Subsequently, a monolayer of polystyrenesulphonate (PSS, average 70 kDa, No. 243051, Merck) was assembled, followed by a monolayer of polyallylamine hydrochloride (PAH, 50 kDa, No. 283223, Merck) and followed again by three washing steps [49]. This procedure is repeated three times to achieve a uniform coating, since this type of polymeric multilayer film starts with an island-like growth based on a charge repulsion between the polymer chains. After this assembly, two bilayers of spherical gold nanoparticles (Au citrate stabilized, 40 nm, No. 741981-25 mL OD1, Sigma, Merck) and PAH were assembled. The final multilayer structure on the silicon dioxide microparticle is chemically described as PEI(PSS/PAH)_3_(Au/PAH)_2_. The polymeric sequence increases the thickness by (16 ± 4) nm, whereby the 40-nm gold nanoparticles increase this structure to (56 ± 4) nm. Owing to the island-like growth, it is unlikely that the gold nanoparticles adsorb in a consecutive manner on top of each other.

Under the illumination of 1550 nm plane wave, the Janus particle would preferentially choose stable orientations, and focus light beam into a PNJ at these stable orientations. For demonstration, we show the focus field by two counter-propagating beams with the orientation of Janus particle at $\left(\theta ,\varphi \right)=\left(85^{◦},156^{◦}\right)$ in Figure 2(b) (corresponds to point E in Figure 1(b)). The Janus particle would concentrate light beam into PNJ on the side opposite to the light source (Figure 2(b)). In presence of both beams, the PNJ would appear on both sides (Figure 2(b)). The orientation of Janus coating is just an example to show the paired nanojets. The orientation of Janus particle is topologically protected to be aligned with the orientation of topological vortices in the streamline map of ***M*** × ***P*** (Figure 1(d)).

To experimentally verify, the synthetic microspheres were suspended in deionized (DI) water (Millipore, 18.5 MΩ). Our observation corroborates that the Janus particle can be pulled individually by a laser beam of either 1.5 μm or 1.65 μm wavelength individually. The synthetic Janus particle is self-powered and moves towards the light source individually in response to the PNJ mediated force. The orientation of Janus coating naturally adjusts such that the nanojet would push the Janus particles towards the light source. Further, the Janus particle moves towards the dominant beam when both beams are switched on. In the presence of right RL of 210 mW, the Janus particles are pulled to the left when the left MLL dominates at a power of 530 mW (Figure 2c). The average speed of the Janus particle labeled in dashed circle in Figure 2(c) is 0.54 mm/s. The Janus particle would move towards right when the power of the MLL is suppressed at a lower power of 140 mW while the power from the right-hand laser dominates at 430 mW (Figure 2d), accordingly the average speed for the particles are 0.14 mm/s.

### 2.3 Competing backaction force on synthetic particles

**Figure 3:**
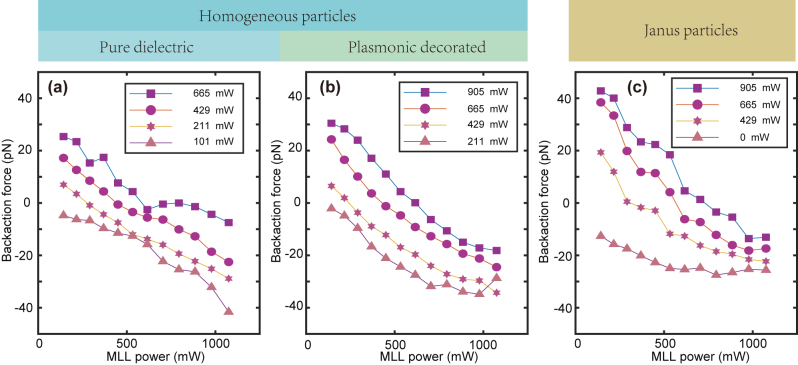
(a) The backaction force for silica dielectric particle. (b and c) The same as (a), but for (b) homogeneously decorated particles, and (c) Janus particles. The RL powers were labeled in respective legends.

The Janus particle can be pulled by the NIR beam with wavelength of 1.65 μm Raman laser. Further, we adopt two beams with different wavelengths from opposite directions to pull the synthetic particles. For easy discussion, the propagation direction of the MLL is chosen as the “+*z*” direction inside the cuvette. The MLL induces a backaction force to the left-hand side (“−*z*”). The continuous RL laser pulls the synthetic particles to the right (the “+*z*” direction). Proper choice of the power ratio results in the balance of the synthetic particles. Figure 3(a) suggests the backaction force for dielectric silica particles with a diameter of 5 μm. As the power of the continuous RL increases, the curves shift upward, and suggest a balance point for proper choice of the power ratio.

In contrast, we also pull the synthetic homogeneous particle. Although the coating is not perfectly transparent to the IR light, the synthetic particles can be pulled by the IR beam. This is further corroborated by pulling the homogeneously decorated synthetic particles with the counterpropagating beams. In the presence of the right RL beam with different strength of laser power (Figure 3b), the backaction force on the particle suggests competing effects between the two counter-propagating beams.

In the absence of the RL, the magnitude of Janus particle speed increases monotonically with the power of the MLL laser (Figure 3c). The competing effect of the two beams takes place once the power of the MLL laser ramps up in the presence of the Raman beam with a fixed power. As the continuous wave Raman laser increases in power, the curve shifts upward. The shifted curve intersects the horizontal abacas with a balanced power of the MLL. Moreover, the power readings for the two beams at balance are non-identical owing to the discrepancy in light absorption in the solvent and the focusing ability of the synthetic particle at respective wavelengths.

The observation on the balance of both the Janus and homogeneous particles using counter-propagating beams also suggests the possibility to control the complex motion of particles using multiple beams from various directions. For instance, three pairs of counter-propagating beams with orthogonal configuration would allow the optical manipulation with full translational degree of freedom.

### 2.4 Force hysteresis on synthetic particles

The dielectric microparticle can focus light into a PNJ, and simultaneously deliver the light energy to the PNJ. The solvent molecules take up the energy with an explosion of heat, and push the microparticles backward. This phenomenon is further corroborated by the experimental observation of the backaction force on silica microparticles. Such phenomenon results in the backaction on the dielectric particle owing to the competition between light absorption and thermal conductivity. The liquid molecules inside the nanojet gain a temperature rise from *T*_0_ to *T*. Consequently, the microsphere experiences a backaction force [41],

(5) $$F=\frac{V_{\text{jet}}\rho_{\text{sol}}}{2\tau }\sqrt{\frac{N_{A}k_{B}}{M_{\text{sol}}}}\left(\sqrt{T}-\sqrt{T_{0}}\right)$$

where *k*_*B*_ is Boltzmann constant, *N*_*A*_ is Avogadro constant, *M*_sol_ is the total number of solvent molecules within the nanojet volume *V*_jet_, *ρ*_sol_ is the mass density of the solvent, and *τ* is the particle response time (on the order of ∼ ms).

The PNJ formed by synthetic particles also concentrates light energy into a narrow region and the energy conversion from light to heat creates a burst on the temperature change. The local temperature *T* is determined by the amount of heat converted from light inside nanojet. Eventually, the heat absorbed by liquid molecules inside PNJ will dissipate and homogenize in the environment and contribute to a lower background temperature *T*_0_. However, there is a time lag between the change in *T* and *T*_0_. The background (local) temperature change originates from the light absorption in the suspension (nanojet). Considering two infinitely close states with low power (*n*) and high power (*m*) (Figure 3a), the system experiences local heating (LH) inside the nanojet and global heating (GH) in the environment. Therefore, the hysteretic response of the backaction force relies on the response difference between the local and global temperature change. The local cooling (LC) is faster than the global cooling (GC). Similar analysis suggests a faster local cooling (LC) than the global cooling (GC) in the power release stage. The magnitude of the backaction force ramps up monotonically with laser power. The dielectric silica particle suggests a similar hysteresis behavior on backaction force as the polystyrene microsphere [42].

**Figure 4:**
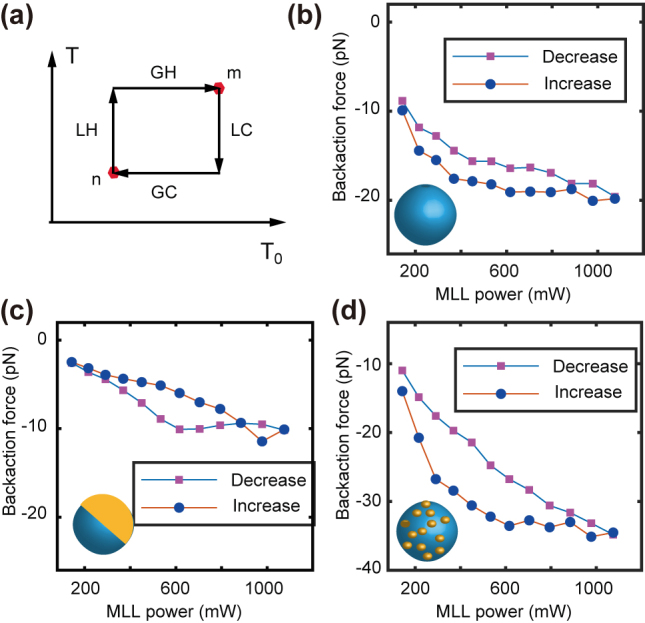
Force hysteresis on synthetic particles. (a) The backaction force was determined by four heating stages. LH, local heating; GH, global heating; LC, local cooling; GC, global cooling. *T*_0_ expresses the ensemble temperature, and *T* represents the nanojet temperature. (b–d) The hysteresis for the (b) dielectric silica, (c) synthetic Janus particle, and (d) homogeneously coated particles.

Analytically, we consider two states with power increase from $p^{\left(n\right)}$ to $p^{\left(m\right)}$ , the temperatures for background and nanojet are from $T_{0}^{\left(n\right)}$ , $T^{\left(n\right)}$ , to $T_{0}^{\left(m\right)}$ , $T^{\left(m\right)}$ . Here, we consider that the Janus particle chooses the orientation at the vortex [Figure 1(d)]. At steady state $T_{0}^{\left(n\right)}<T_{0}^{\left(m\right)}$ and $T^{\left(n\right)}<T^{\left(m\right)}$ (Figure 4a). The nominal force at equilibrium would be, $F^{\left(i\right)}=\sqrt{T^{\left(i\right)}}-\sqrt{T_{0}^{\left(i\right)}}$ , where *i* = *m*, *n*, indicates two representative states. Here, the same constants in Eq. (5) are omitted for concise expression. Practically, the backaction temperature increases much slower than the temperature inside the nanojet, therefore the actual force approximates, $F_{\text{hys}}^{\left(m\right)}\approx \sqrt{T^{\left(m\right)}}-\sqrt{T_{0}^{\left(n\right)}}$ .

The difference, $F_{\text{hys}}^{\left(m\right)}-F^{\left(m\right)}\approx \sqrt{T_{0}^{\left(m\right)}}-\sqrt{T_{0}^{\left(n\right)}}>0$ , implies that the backaction force increases faster than the nominal force. The hysteretic force $F_{\text{hys}}^{\left(m\right)}$ is greater than the nominal value $F^{\left(m\right)}$ when the injected power ramps up. Figure 4b suggests a hysteretic force loop for dielectric silica particle when the injection laser power decreases after a continuous increase in the power. Such observation is consistent with our previous report on polystyrene spheres and biological cells [42]. We further show that such hysteresis loop does exist in the force curve of synthetic particles. Figure 4c shows the force hysteresis for the synthetic Janus particle. Apparently, the hysteresis is highly suppressed preferentially due to the anisotropic coating. The backaction force for homogeneously decorated particle also suggests an augmented hysteresis loop (Figure 4d). Our observation on the hysteretic force response for synthetic particles further confirmed that the backaction force is jointly determined by the average laser power and the previous status that the particle suspension was experiencing.

The synthetic particle suggests hysteresis on the balanced force as observed on dielectric particles. The hysteresis originates from the response delay between the global temperature rise and the local temperature rise inside the nanojet. Moreover, the synthetic particle allows more ingredients and powerful properties to strengthen the application, for instance, improved magnetism and hydrodynamic properties combined with the backaction force, may potentially be applied for biomedical micromanipulation, and microsurgery. The understanding of the backaction force on synthetic particle may also boost the application to manipulate multiple particles with complex geometries, e.g., the biconcave red blood cells [50]. Combined with up-conversion rare earth nanoparticles, the swimming synthetic particle can also be used for fluorescence imaging using the infrared beam as excitation, offering the possibility to report the particle location [12].

## 3 Conclusions

In conclusion, we report on the all-optical parallel pulling of synthetic particles using PNJ mediated backaction force. Specifically, the optical pulling of the Janus particle is enabled by topologically protected PNJ. Due to reduced transmission, the homogeneously decorated synthetic Janus particle can also form PNJ and mediate backaction force. These have been corroborated by experiments that both the Janus particles and the homogeneous particles can be pulled by non-resonant light. We observed hysteresis on the backaction force of those synthetic particles. The backaction force on those synthetic particles can also be balanced by dual counter-propagating beams under proper choice of the ratio of the beam power. These synthetic microparticles were once thought to be unable to form time-invariant PNJ. Counterintuitively, our research elucidates that with specific orientations protected by topology the synthetic particle can form stable PNJ. Our research not only provides insights into the fundamental mechanism behind the formation of PNJ with synthetic particles, but also highlights the potential to utilize the PNJ-mediated force to sort broadband microparticle species, paving the way for novel applications in optically controlled nanomotor and drug delivery.
